# Genetics, emotion and care: Navigating future reproductive decisions in families of children with rare genetic conditions

**DOI:** 10.1111/1467-9566.13854

**Published:** 2024-10-12

**Authors:** Catherine Coveney, Basma Salem

**Affiliations:** ^1^ Department of Criminology, Sociology and Social Policy School of Social Sciences and Humanities Loughborough University Leicestershire UK

**Keywords:** care, disability, genetic subjectivity, human reproduction, rare genetic disease, reproductive decision‐making, selective reproductive technologies

## Abstract

Little is known regarding the future reproductive decision‐making of parents of children with rare genetic conditions. Our research draws on data from an online survey and qualitative photo‐elicitation interviews with families living with Noonan Syndrome. We demonstrate how genetic knowledge and prenatal genetic testing become embedded in reproductive practices. Yet the idea of using selective genetic technologies to influence reproductive outcomes remains highly emotive. Our analysis reveals that for these parents, the rationalities of reproduction, although technologised and biomedicalised, remain centred on caring for their disabled child. Genetic subjectivities become entangled with responsibilities of care‐giving and emotion tied to the realities of living with disability. We argue that for these parents, reproductive decisions are *relational and affective*, situated within families and communities and shaped by access to emotional, financial, physical and temporal resources. Our findings provide new insights into the ontologies of selective genetic technologies and reproductive governance in families living with disability.

## INTRODUCTION

Little is known about how having a child diagnosed with a rare genetic condition impacts on parents’ future reproductive decision‐making. Our analysis demonstrates how ontologies of genetic diagnostic tools and selective technologies are embedded in different logics. It reveals tensions between the dominant medicalised and politicised ontology of these technologies as a way to prevent transmission of genetic conditions and parents’ ontologies of reproductive genetic technologies as informational tools that can be used to prepare for the birth of an affected child. We show how, in parents’ reproductive practices, their genetic subjectivities become entangled with the politics of care‐giving and emotion tied to the realities of living with disability. We argue that reproductive decisions are *relational and affective*, being situated within families and communities as well as physical, economic, temporal and emotional resources that enable and constrain the reproductive decisions families living with disability are able to make.

The original contributions of this article are twofold; (i) to fill a gap in the literature on how the lived experiences of parenting a child with a rare genetic condition interacts with future reproductive decision‐making and (ii) to bring social scientific literature on selective reprogenetic technologies and disability studies on parenting into conversation with one another. Therefore, this article responds to calls for increased dialogue between medical sociology and disability studies (Thomas, 2021) and contributes to building intellectual solidarities that can strengthen scholarship and influence policy and practice.

### Noonan syndrome

Our research focuses on families living with one rare autosomal dominant genetic condition, Noonan syndrome (NS). First characterised in 1964 by Dr. Jacqueline Noonan, an American cardiologist, it is one of the lesser known ‘rare diseases’ with an estimated prevalence of around 1:2000 live births, similar to rates of cystic fibrosis in European populations. Noonan syndrome is medically complex and associated with a wide range of health issues including cardiac, lymphatic, gastrointestinal, neurological, musculoskeletal, kidney, blood, vision and hearing, growth and development, sleep, childhood cancer, mobility and pain (Roberts, 2022). Psychological, educational and behavioural issues are also common, with many individuals experiencing speech and language difficulties, neurodivergence, specific learning difficulties and disabilities and requiring special educational support at school (Coveney & Lambert, 2023). How people are impacted by NS is variable; some are more mildly affected whereas others have severe health issues that can be life‐limiting.

Today, NS is typically diagnosed in early childhood via a specialist gene panel. Genetic diagnosis can confirm the presence or absence of one or more pathogenic gene variants across at least 14 known genes (Roberts, 2022). It is estimated that around 20%–40% of cases are inherited, with the rest being ‘de novo’, meaning that they occur spontaneously (Zenker et al., 2022). However, epidemiological data is lacking making true prevalence rates unclear. As we learn more about the genetic profile of NS, and genetic testing becomes more accessible, genetic diagnosis of NS is taking place at earlier points, including prenatally. Should a known pathogenic gene variant be detected during pregnancy, parents are usually given the option to terminate the pregnancy. Wider accessibility of genetic testing is also leading to the identification of an increasing number of cases of adults being diagnosed, often after the birth of a child exhibiting a more severe clinical presentation.

### Reproductive decision‐making and reprogenetics

The politics and practices of reproduction are not only central to our social lives, but a central concern in social theory, providing vital insights into how our societies are organised, our shared cultural values and visions for the future. There is a wealth of scholarship that analyses how scientific, medical and technological innovations have shaped the landscape of human reproduction and reproductive health over the past 30 years. Reprogenetics is a term used to describe the combination of genetic tools and reproductive technologies (de Melo–Martin 2022) such as IVF, pre‐implantation genetic diagnosis, embryo selection and gamete donation. Within dominant reproductive imaginaries, the ‘valued reproductive’ citizen (Saunders, 2021) follows appropriate reproductive trajectories imbued with moralised notions and norms about the right kinds of people, having the right number and *right kinds of children* (Wahlberg & Gammeltoft, 2018) at the right time and within the ‘right’ family formations (Baldwin, 2017).

Although reprogenetic technologies are often presented as tools for ‘colonising the reproductive future’ (Myers, 2014), the idea that we, as individuals, are free to choose our own reproductive paths has long been critiqued. The recognition that social inequities and material condition’s structure reproductive decisions sits at the heart of reproductive politics (Hall, 2023). Thus, notions of reproductive ‘choice’ are recognised to privilege white, middle‐class women who have the financial, cultural and educational capital to choose between different reproductive options (Roberts, 2015). In short, the reproductive ‘choices’ available to us are stratified across several intersecting factors, including but not limited to ethnicity, class, age, disability status and geographic location. Furthermore, true reproductive freedom involves not only the right to have children and the right to abortion, but also *the option to carry a child with disabilities to term* (Roberts, 2015) and *the right to raise children with dignity, regardless of their health or disability status* (Jaman, 2015). However, within state sponsored screening programmes, typically, healthy pregnancies (Michie & Allyse, 2022) are positioned as desirable while implicitly suggesting that selective termination is “the right intervention” or a “a solution to disability” (Bryant & Shakespeare, 2022:48).

Recent work shows how newer selective reproductive technologies are also imbued with disease and disability‐free imaginaries (Herbrand et al., 2023) being positioned as a way for prospective parents to avoid passing on hereditary genetic conditions to their future children (Wahlberg & Gammeltoft, 2018). It is clear then, that some reproductive futures are valued and prioritised, empowering particular groups of people to reproduce, and certain types of babies to be born, while others are devalued (Ginsburg & Rapp, 1995).

With the rise and routinisation of genomic medicine in reproductive healthcare, parents of disabled children are among those most likely to encounter genomic medicine during subsequent pregnancies. This includes during the counselling, planning, prenatal and/or postnatal phase. These parents must contend with complex genetic information, and make decisions, often based on risk factors with uncertain outcomes (Boardman, 2017; Kelly, 2009) within dominant medical framings of disability as a problem to be avoided.

How parents who already have a child with a rare genetic condition navigate these complex reproductive decisions is largely unexplored in the literature. There is a small body of work that addresses this specific group (Shorey, 2023), with even fewer studies employing a qualitative sociological approach (Fearon, 2023). The available literature suggests that often parents of children with genetic conditions do not go on to have further children (Kelly, 2009; Raspberry & Skinner, 2011). These parents may feel a sense of ‘genetic responsibility’ (Boardman, 2017), that is, as responsible parents, they should prevent the transmission of disease‐causing genes to future generations by either not having (more) children or engaging with selective reproductive technologies to manage genetic risk (Kelly, 2009).

Expanded notions of genetic responsibility, however, explicate how genetic knowledge is but one factor shaping reproductive decisions and ideas of being ‘genetically responsible’ can be enacted in competing ways. For instance, in Raspberry and Skinner’s (2011) study of families with known genetic risk, those who did go on to have more children prioritised moral frameworks that valued parenthood and disability. For these parents, their sense of ‘doing the right thing’ (2011:429) was entangled with the rejection of distinctions between people with disabilities and those without. Likewise, parents in Mendes et al's (2021:1480) study made informed decisions to have ‘an at‐risk’ child, valuing parenthood, family relationships and life above questions of genetic disease. Furthermore, Boardman (2014) draws attention to how lived realities of disability and experiential knowledge of a condition shape reproductive decisions, including how people justify their use/non‐use of selective reproductive technologies.

### Parenthood, disability and care

The experiences and realities of parents managing complex care for their children with rare genetic conditions is another area that has received limited sociological attention (Currie & Szabo, 2019) with disability too often “at the fringes of sociological thought” (Thomas, 2021, p. 454). What research there is indicates that parents can have substantial responsibilities for providing complex care at home, becoming nurses, therapists and teachers to their medically complex child over extended periods (Ryan and Runswick–Cole 2008). Research highlights the everyday challenges of care‐giving families face including social isolation, exclusion, stigma, navigating fragmented healthcare systems, higher incidence of relationship breakdown and financial and emotion strain. This is compounded by a lack of public and medical understanding of these realities and insufficient support (von der Lippe et al., 2022; Currie & Szabo, 2019; Coveney & Lambert, 2023; Loft, 2011).

Looking to the growing body of work within disability studies on parenting disabled children, we find rich accounts of how parents deal with day‐to‐day complex medical care, educational decisions and take on advocacy roles (Blum, 2015; Thomas, 2021). Often the care work parents of disabled children undertake is invisible as it becomes incorporated into daily routines and practices (Coveney & Lambert, 2023). The realities of the child’s disabilities, illnesses and/or impairments see the extraordinary—such as life and death decisions—becoming normalised (Jackson, 2021) and realities of care‐giving become folded in with what Mattingly (2014) calls the “humble moments of everyday life” among people who are trying to create a good and happy life for their family in the face of uncertainty and unpredictability (Baumbusch et al., 2019). Nevertheless, parent’s accounts of parenting disabled children are often celebratory, centring experiences of joy, pride, value, ‘normality’ and happiness. Thus, compelling us to reimagine dominant cultural scripts of disability as tragedy, suffering and hardship (Thomas, 2021b).

In the remainder of this article, we draw on empirical data to explore how caring for a child diagnosed with NS impacts on parents’ future reproductive decision‐making. We ask: how does genetic diagnosis of their child impact on parents own genetic subjectivities? What role is given to reproductive technologies and genetic medicine in future reproductive decision‐making? How is the lived experience of caring for a disabled child entangled in these processes?

## METHODS

The project received ethical approval from Loughborough University ethics committee in April 2022. An anonymous online survey was distributed via social media to two NS‐specific Facebook groups and by email to members of the NS Association UK via their mailing list, between June and November 2022. The recruitment materials for the study invited individuals and family members (18+) living with NS to participate. The definition of ‘family’ was kept intentionally broad to be inclusive and capture the diverse networks of care and kinship that exist in contemporary Britain. Survey questions were designed to gather information on (i) the characteristics of respondents, (ii) medical, psychological and social impacts of living with NS and (iii) perceptions and sources of social and emotional support. The survey was completed by 67 individuals, the majority of whom were parent–carers of children with NS (*n* = 58). Survey respondents predominantly identified as being from a white ethnic background (90%) and as female (80%) with a mix of social class indicators.

The survey was also used to recruit participants for the interviews. Sampling for the interviews was open and opportunistic. All survey respondents were invited to leave their contact details if they wanted to participate in the interview. All 35 participants who expressed an initial interest were invited to attend an interview over Microsoft Teams that fit around their schedule. However, recruitment was challenging, and many participants dropped out of the study at this stage due to their own or their child’s health and care needs taking priority and difficulty scheduling a time for the interview.

Follow up interviews were conducted with 18 parent–carers between September 2022 and October 2023. Participants chose who was present during the interview and whether they wanted to be interviewed individually or as a couple/family group. Three interviews were carried out as couples (all mother/father dyads). The couple dynamic shaped the information that was offered to some extent, particularly how candidly participants spoke about their own relationships and the gendered division of care. When interviewed together, couples tended to speak relatively equally and offer shared perspectives to provide a balanced view of parental roles and responsibilities. They helped each other to recall details of specific experiences which added depth to the data.

Furthermore, in several of the individual interviews another family member (e.g. child’s grandparent or participant’s partner) was present in the room but did not participate in the interview other than providing supportive comments or reassurance when these were sought by the participant. During three interviews, children were present for a time, entering and leaving the room in their home that their parent was video calling from. Although children spoke to their parents and were introduced to the researcher during these times, these exchanges were not included as research data. The presence of children in the interview space did impact on interview encounter. We observed that participants often changed the tone and topic of conversation while their children were present to focus more on positive aspects of life with NS rather than its challenges or their own feelings.

Interview participants were aged between 29 and 76, with an average age of 48. All interview participants identified as White British and were either mothers (*n* = 13) or fathers (*n* = 5) of children with NS. Of these, seven participants worked in professional occupations (3 part‐time, 4 full‐time), three in technical or manual occupations (all part ‐time), 1 was retired with the remaining participants identifying as full‐time carers and not in paid employment (*n* = 7). Thus, we acknowledge that despite our efforts to recruit a diverse sample, our data do not represent a universal experience of living with this rare genetic condition.

Seventeen survey respondents had a diagnosis of NS themselves, with eight respondents also having one or more children diagnosed with NS (Coveney & Lambert, 2023). We were not always able to tell from the survey data whether their diagnoses occurred prior to or after having children. We used the interviews to explore the circumstances surrounding reproductive decision‐making in NS for parents (with and without a diagnosis themselves) in more depth. Other salient themes in the survey data informed the follow‐up questions we asked in the interviews.

The interview process was centred around storytelling via photo‐elicitation. Participants were asked to submit up to three photographs in advance of the interview to represent what living with a rare genetic condition meant to them. The photo‐elicitation method was selected to give some control over the interview process to participants, allowing the careful consideration and broaching of sensitive topics and to help participants emotionally prepare for the interview (Aldridge, 2007; Papaloukas et al., 2017). Participants were asked follow‐up questions, in no order, relating to the content of their photographs and around the following themes of diagnosis, care‐giving, relationships, reproductive decision‐making and social and emotional support. We found that the photographs provided a mechanism for participants to express complex emotions visually, which are often difficult to articulate in words, and discuss private moments that may otherwise have been missed, resulting in rich and detailed accounts (Fearon, 2019).

Interviews were recorded, transcribed and coded in NVivo 14. Interview data and answers to open text questions on the survey were coded using a mix of open coding and theoretical coding. We took an interpretative sociological approach to develop patterns of meaning across the dataset. Reflexive thematic analysis (Braun & Clarke, 2019) was used to generate themes. This involved the careful reading and re‐reading of interview transcripts by both authors individually, followed by the systematic analytical process of grouping together data extracts based on their main topics, development of a coding frame and connection of congruent codes together, with discussion between authors to generate, develop and review themes.

For the purposes of this article, data relating to genetic diagnosis and testing, reproductive decision‐making and care ‐giving will be discussed. Although informed by the survey analysis, data excerpts presented in the article are predominantly drawn from the photo‐elicitation interviews. In the following section we discuss three themes relating to (i) genetic subjectivities and engagement with reproductive technologies and/or genetic medicine, (ii) choreographies of care and (iii) emotion and care.

While the gendered dynamics of care was a significant theme in the data overall, there was no discernible gendered difference in how mothers and fathers spoke about their reproductive decisions. This could be because of the relativly small number of fathers interviewed, and that three fathers were interviewed with their spouses, during which they provided shared accounts that were aligned and consistent with one another. Thus, we refer to participants collectively as parents throughout the article.

## FINDINGS

### Genetic subjectivities and engagement with genetic medicine

For many participants, their child’s diagnosis of NS substantially impacted their future reproductive decisions. About half of our participants underwent genetic testing at the time of their child’s diagnosis and were informed that their child’s NS resulted from a spontaneous mutation. A smaller number had received a genetic diagnosis of NS themselves after their child was diagnosed. In all but one case, this involved both parents undergoing genetic testing. The only exception was Sarah who was a single parent. She had reached out to her ex‐partner after their son’s diagnosis, who did not want to be tested:

Sarah When (child) had his genetic testing, I was tested and my mum was tested, we went further down the family tree and we both came back as non‐carriers. They asked if the father would be interested in being tested ‐ I did contact him and ask, and he said no. I think it was about his “perfect life”, I don't think he would've been able to cope with that […] He literally just washed his hands of the whole situation. It was a case of “he's your baby, not mine. You deal with it”.

This new biogenetic knowledge impacted on parents own genetic subjectivities, prompting a genetic re‐telling of their family history as they came to think of themselves as vehicles for transmission of the ‘mutated gene’ (Jenny) or ‘non‐carriers’ (Sarah). As illustrated in Stacey’s account below, this new genetic understanding of their family often played a pivotal role in shaping future reproductive decision‐making:

Stacey It was explained to us that if she has a spontaneous mutation, there's not any chance that the next one would have because she didn't inherit it from us, so I didn't worry that there was a risk that we would have another one [with NS], although they did monitor when I got pregnant.

Some participants, like Marie, expressed that having a child with NS strengthened their prior decision not to have further children. Others stated that their existing preference for a smaller family or their age was the deciding factor.

Marie We had this one child, we didn’t view it as the beginning of having a big family. When we did go through these issues, it justified my thinking, I thought “I don’t want another one, I’m not having another one…. Here’s a doubly good reason. She’s our focus. End of story”.

Other families either were not offered or decided not to engage with genetic testing, rejecting these new regimes of genetic surveillance. An exemplar here is Lisa who has a 9‐year‐old daughter clinically diagnosed with NS. Lisa described a difficult pregnancy during which she underwent various medical tests including an amniocentesis and karyotyping to check for potential chromosomal issues with the foetus. The possibility her baby could have NS was not raised at this point; the genetic tests came back negative for a range of other possible genetic conditions. Her daughter received a clinical diagnosis of NS aged two. As a family, they decided not to undergo further genetic testing postnatally because they did not think this would change anything for their daughter. As a result, they are unsure of the heritability of NS within their family. This uncertainty, alongside imaginings of a future pregnancy that is also difficult and medicalised, influenced their decision not to have any further children. We can see here how positive postnatal genetic testing can produce conditions for future engagement with selective reprotechnologies should these parents decide to have more children:

Lisa We decided not to have another one. A part of that was because when we were with the professor, she said that we’d have to be tested, we’d potentially have to have IVF, and then they could choose the embryo that hasn’t got it from us, and we just thought [pause] she’s enough to make‐up for three kids sometimes.

Typically, participants had some awareness of the reprogenetic technology available to them, should they decide to have another child, including genetic testing, engagement with reproductive technologies (IVF and embryo selection) and genetic screening during pregnancy. Participant’s relationships to these technologies were complex and varying. The idea of ‘selecting’ against another child with NS through engagement with reprotech was a deeply emotive issue. Many parents, like Samuel, likened the idea of selecting against NS to the rejection of his existing child:

Samuel Would I have another child with NS? It’s a big consideration, (child) needs that extra level of support, she is an amazing kid […] why would I not want more (child’s name)? ‘cause she’s incredible. I’ve learnt so much from her Noonan’s Syndrome, it’s made me a better person in many ways.

Participant’s children were impacted by NS at different severities. At one extreme, Laura’s son had passed away as a teenager due to severe cardiac complications. At the other end of the spectrum, Stacey was the only participant to describe her child’s symptoms as mild. Despite this, she told us how her child had experienced cardiac, growth and development, visual impairment, behavioural and mental health issues and had learning disabilities leading to special educational needs. The perceived severity of the syndrome did not appear to have a significant bearing on whether the participant had gone on to have subsequent pregnancies or not. Stacey, Richard and Sarah had all gone on to have more children, with Samuel considering the possibility in the future. Furthermore, complicating the rhetoric of reproductive choice which silences experiences of infertility and miscarriage, it is important to note that Laura had tried to conceive again when her son was young without becoming pregnant. Dave and Michelle also disclosed that they had conceived again and had experienced miscarriage.

One participant, whose child was diagnosed as a teen, shared that she would have opted for the termination of pregnancy had she received the genetic diagnosis prenatally. Yet, in retrospect she was glad she was unaware, as she loves her child deeply. Typically, most parents were not fearful of having another child with NS and did not think of their reproductive decisions in terms of genetic responsibility or risk management. As we can see below, although initially framing her discussion in terms of genetic risk, Jenny quickly corrects herself, reframing the biomedicalised language she had been provided with away from ‘risk’ to ‘percentage chance’:

Jenny I have the mutated gene, the faulty gene […] when we did think about having more children, we spent quite a lot of time with our geneticist, working out sort of risk, not risks, but percentage chance of it happening again…it’s 50/50.

Furthermore, accounts like Richard’s highlight conflict arising from divergent ontologies of genetic diagnosis and selective reproductive technologies. Despite Richard and his partner testing negative for NS at the time of their daughter’s diagnosis, they still grappled with uncertainties about the health of their future child. After several miscarriages, they conceived again and were offered an amniocentesis and genetic testing for the foetus via the NHS, which they declined. Richard described feeling pressured by medical professionals towards selective termination should the tests come back positive for NS. Scared by the trauma of several reproductive losses, he and his partner remained anxious and uncertain about whether their baby would be born healthy. Yet they rejected the medicalised ontology of these practices as a way to prevent disability. Instead, they paid privately for Non‐Invasive Prenatal Testing (NIPTs) to gather more knowledge about the potential future genetic health of their unborn child to help them prepare for what was to come:

Richard We had NIP testing [we were] just on edge constantly and fearing that things were gonna go wrong. It's such a tricky decision […] there's no guarantees, even if you do all the testing, doesn't mean everything's gonna work out right.

The analysis shows that for these parents, the rationalities of reproduction readily become technologised and biomedicalised as their bodies become subject to new regimes of genetic surveillance. Biogenetic knowledge and new genetic subjectivities become embedded within future reproductive decisions and can impact on reproductive governance as parents contend with the genetic ‘risk’ factors they have been presented with. However, the role of genetic subjectivity and reprogenetic technologies in parents’ accounts of their reproductive decision‐making was always entangled with, and often overshadowed by, focus on caring for their existing child with NS.

### Choreographies of care

Caring for a child with a multi‐system syndrome like NS means juggling complex medical, social, educational and emotional needs. Parents explained how their days were dominated by taking their child to frequent medical appointments, especially throughout their early years, organising medical care, administering various medicines and other therapies (including speech and language therapy, physiotherapy and occupational therapy exercises) at home, preparing special diets and assisting with personal care. In addition, they spent a lot of time researching their child’s various health conditions and symptoms, advocating for them, teaching and guiding them and helping them to build friendships as well as playing with them, nurturing them, listening to them and loving them.

Care is intimate and relational, involving human intimacies such as touch, massage, holding hands and hugs as well as non‐human technologies, medicines and medical devices–pills, needles, feeding tubes, liquids and prescription diets. Care can become all consuming, where parents often felt their lives revolved around their child’s health needs. The intensive nature of caring, in some cases over decades, leaves little time for self‐care particularly if parents do not feel adequately supported. In the extract below Meg gives us a glimpse into her life as a parent–carer for her six‐year‐old daughter with NS. She talks about feeling exhausted yet having to fight all the time to be heard and to be listened to, so her child gets the care she needs:

Meg She's been to hundreds of appointments, she's under a lot of different consultants […] There's never a doctor that's making sure everything's happening as it should. You are the glue that has to put all this stuff together ‐ do the research, make the suggestions, you become the expert in your own child. The pressure of that is enormous and it’s impacted every fibre of my being, my career, my sleep, my health […]. The stress it puts on you, on your relationship […] It's like you're on a roller coaster that you can't ever get off […] it never feels like enough hours in the day.

Lack of support was a dominant theme in both the interviews and survey data and was raised by every interview participant and survey respondent alike. The data indicate the extent to which structural constraints add additional challenges for families living with disability and shape their experiences of care. For instance, survey respondents described limited or no access to childcare provision, expressing difficulties finding childcare providers that would accept their child’s complex health needs:

Unable to leave my baby in care due to his needs. Low immunity causes hospitalisations, so Dad also keeps needing to stop working and help so lack of money coming in.

(Survey respondent 21, individual with NS who has a child with NS, aged 43)

Several participants, like Jenny and Helen, felt that community groups were not set up to meet their child’s needs:

Jenny There was a birth and baby cafe down the road, but it was quite painful to go there because all of the kids there were typically developing. The normal baby groups weren't really set up for us.

Helen We didn't get to go to many baby groups, I wasn't having the same experience as all those other parents. And that can feel quite isolating.

This often led to financial strain as parents felt they had no option but to leave paid employment, or were not able find flexible paid employment to fit around care‐giving:

Being unable to return to work as [child] is not able to manage childcare for long also results in additional financial stress and worry about my future career prospects. I worry about finding employment that will accommodate my child’s numerous appointments. We are unsupported by our family as they don’t feel that they can manage my child.

(survey respondent 14, Mother aged 42)

Furthermore, many respondents described having to fight for educational support for their children. While a small number had secured an Education and Health‐Care Plan (EHCP) or a place in a specialist educational setting, typically participants described an arduous process of getting their child’s educational needs recognised and supported:

We had to fight for an EHCP […] it being years of battling and being at crisis point with our children having extreme anxiety as things have had to get to breaking point before anything is done.

(Survey respondent 11, Mother aged 48)

In terms of the provision of social care, those with now adult children often expressed fears about their future and who would look after their child when they were no longer able to. Social services were described as ‘inadequate’ and some families had difficulties accessing governmental benefits or other assistance, such as a blue badge for access to disability car parking, despite their child using a wheelchair or having high mobility needs:

Total ignorance of authorities in recognising NS and little support to make life better. Cannot even get a parking badge from our local authority, our daughter has three prosthetic valves.

(Survey respondent 3, Father, aged 68)

The implications of these challenges were evident when considering whether to have more children. Within this context, parents imagined difficulties in being able to provide necessary care should a future child also have complex health needs and require as much extra time, care and support as their existing child does. The practical difficulties of raising a child with NS, such as restless nights and frequent medical appointments, the resulting temporal constraints, lack of external support, emotional labour and impacts on parents’ own physical and mental health were strong components of their decision‐making. Participants often described feeling they were already at ‘breaking point’:

Richard It’s not the fear of having another child with additional needs. It's whether you could give them the same level of support because already you are almost at breaking point with one … you know the amount of help and care that's required and how these babies thrive through the additional care that you give, and if you can't give that how would you forgive yourself? […] It’s had a massive influence on our family 'cause we wanted another child but… there's financial implications. There's care implications. You just can only do so much and there's very little support.

Like Richard (above) and Laura (below), many of the parents we interviewed feared that if they had another child, their attention and physical, financial and temporal resources would be stretched too thin, compromising the quality of care and support they would be able to provide to their existing child(ren):

Laura I would have liked more children […]. But I was always a bit half‐hearted about it. It wasn't so much about the diagnosis, it was just more sort of practical things, because right from the beginning, it was clear that (child) needed a lot of extra help. I couldn't see how it would be possible to manage with more than one, even if they didn't have Noonan’s.

However, it was also apparent that the contours of care‐giving shift over time. For many of the families we interviewed, the early years were identified as the most challenging period. Once care‐giving had been normalised within the family, families had ‘learnt the ropes’ and some of the early difficulties associated with NS, particularly around feeding and developmental delays had begun to resolve, the perceived intensity of care‐giving could lessen. In these instances, imagining the possibility of caring for another child alongside meeting their existing child(ren)’s needs became more of a realistic possibility for some families:

Stacey We were certainly like ‘we're not gonna try and have another one yet because we need to see how she is. How much effort we need to put into her? Will we have time to deal with another one?’ We were very unsure […] Once we realized her heart problem and other physical problems were resolving I was like, ‘yeah, I think we can handle another one’.

To summarise, it was evident that future reproductive decision‐making for families already living with a rare genetic condition is relational, situated within wider networks and relationships and the complex choreographies of care they are made up of. Reproductive governance is influenced by families’ lived experiences of disability, including structural constraints and material conditions, and is shaped by whether parents feel they have the physical, temporal and financial resources to care for another child.

### Emotion and care

Finally, we discuss how emotions stemming from the lived experience of parenting a child with a rare genetic condition interact with genetic subjectivities and the sociomaterial aspects of care outlined in the previous section. As mentioned above, some families, like Lisa’s decided not to have more children, prioritising the health and wellbeing of their child with NS and existing siblings. These decisions had a deeply affective dimension. Rather than being shaped solely by ideas of genetic responsibility, they were tied to the intimacies of care‐giving and parents felt obligations and responsibilities to not only meet their child’s needs but be attentive, compassionate and nurturing. Several interview participants talked about feeling guilty for not having enough time for their existing children and sometimes struggling to meet their emotional needs. We can also see this reflected in the survey data:

Guilt You feel as if you are constantly rushing to ensure all treatment and medication is administered on time at the expense of playing, reading. You never feel you are ever present as you are thinking of the challenges on the horizon. [Survey respondent 1, Father aged 42]

They feared that having another child would squeeze their time, attention and emotional resources even further.

For others, reproductive decisions were entangled with complex emotions tied traumatic experiences that had faced during pregnancy, birth and the early years of parenting a baby with complex health needs. Participants described their experiences of complicated pregnancies, difficult births, pregnancy losses, life‐threatening health complications, emergency surgeries, hospitalisations and witnessing their child in pain. They told us stories of medical trauma, delayed diagnosis, fighting for medical recognition and lack of support and explained how these experiences had negatively impacted on their own health and wellbeing and on their relationships. Although she had already decided on her family limit, Helen describes how early traumatic experiences caring for her 2‐year‐old son with NS, who had had cardiac surgery twice along with repeated hospitalisations, cemented her decision not to have any more children:

Helen They nearly killed us both in his birth. [As a newborn] he wouldn't feed, and it was just a nightmare … he used to just scream permanently. I was at my wits end. All he did was vomit and cry with this horrendous, “I'm in pain, help me somebody, please stop this” cry […] nobody was listening to me saying there is something wrong with my baby […] I knew then that was me done … because I wouldn't risk going through this again.

Jenny tearfully described a difficult pregnancy which took its toll on her health and wellbeing. She had spent a lot of time in hospital and considers how, if she were to experience that again in a future pregnancy, it would impact on her ability to care for her daughter with NS. She feels it would be unfair to her existing child if she were not able to meet her needs:

Jenny It was a really difficult decision to not have more children because I did want a sibling for [child] and [partner] always wanted more children. The main reason for me was I felt so disingenuous to talk to [child] almost like saying, well, I wouldn't have another one of you. And that certainly isn't the case. What I've felt was I could not have been in hospital again all that time and having her at home without me, it would be completely unfair to her.

Stigma, both perceived and felt, was a strong theme in both datasets. Most respondents described encountering negative attitudes towards disability in the workplace, school and wider community. Participants, like Sarah in the data extract below, recounted traumatic experiences of bullying and social isolation that their child had experienced and noted significant impacts on their child’s mental health and wellbeing.

Sarah He was off school for nearly six weeks [provides specific details of verbal and physical abuse]. He wrote in his diary one night that he wanted to die […] we had a meeting to try and get him into a special school, he's been put on a two‐year waiting list. […] It makes you feel sick. There's nothing you can do. There is nothing out there. There's no help for them at all as children or you as parents. Everything you try and fight for, you just have to wait so long. We've been trying to get a community paediatrician for five years and every single time, we’ve had refusal from them which I think is actually disgusting when there's a child that needs help. Next, I will have to write to the MP. It's absolutely exhausting. It's emotionally exhausting having to fight for everything all the time.

Like Meg, participants rooted their reproductive decisions within the emotional aspects of the realities of living with disability and expressed fears about having another child who might have similar experiences of the world:

Meg It would have definitely been a consideration if we were thinking of having a third because even though (child) having it, it's not a gene fault that me or my husband have because we were tested… But I guess there'll be a level of fear… if it were to happen again, just knowing how hard life can be for (child) and just seeing that first hand.

In sum, emotions tied to experiences of parenting a child with complex needs and the realities of living with disability, coupled with a desire to prioritise the needs of their existing children can impact on reproductive decision‐making in significant ways. We can see how reproductive decisions are both *relational and affective*, that is, situated within families and communities and shaped by access to a range of physical, economic, temporal and emotional resources.

## DISCUSSION

Our analysis focuses on future reproductive decision‐making by parents who already have a child with a rare genetic condition. Our findings add richness to the limited body of literature on how the lived experience of having a child with a rare genetic condition shapes the complex dynamics of reproductive decision‐making. We show how parent’s decisions on whether to have more children are entangled within an amalgam of biogenetic knowledge, choreographies of care‐giving and emotion situated within their family context. We argue that although the rationalities of reproduction become technologised and biomedicalised when living with a rare genetic condition, for these parents, reproductive decision‐making, and the possibility of future children remain centred on the realities of—and emotions connected to—caring for their disabled child.

Living with rare genetic disease inevitably raises the question of whether and how to proceed with further pregnancies. Existing research indicates that parents of disabled children approach future pregnancies with caution, not taking for granted that a future pregnancy will result in the birth of a healthy baby (Shorey, 2023). Engaging with reproductive technologies can tie into a curative imaginary (Kafer, 2013), a way to avoid the birth of a disabled child (Kelly, 2009), within which disability is often positioned as negative, burdensome, tragic and limiting (Roadhouse et al., 2018). Reproduction, in Kelly’s (2009) study was viewed as inherently risky and the birth of children with disabilities and impairments as negative or undesirable outcomes. Previous research suggests that genetic testing places genetic responsibility predominantly on women, who are positioned as primarily responsible for their family’s health. This includes the moral imperative for women to engage in genetic testing and risk management to potentially prevent the transmission of genetic conditions to future children (Hallowell, 1999). In our study, however, this gendered dynamic was not typically observed, with both parents tending to either undergo genetic testing or not. In relation to ideas about genetic responsibility, like Mendes et al. (2021), the parents we interviewed did not necessarily view the transmission of genetic condition itself as a ‘risk’ they would wilfully avoid in the future. While some parents alluded to ‘suffering’ (Boardman, 2017) and not wanting themselves or their future child to go through what their disabled child has endured, most participants centred their responsibilities and obligations to be good carers for their children as central to their reproductive decisions.

The data show that, in the contemporary context, for many parents who already have a child diagnosed with a rare genetic condition, genetic knowledge has become a part of their social fabric. They have already come into contact with a range of prenatal and antenatal genetic technologies, geneticists and genetic counsellors with many undergoing genetic testing themselves before planning or even considering possible future pregnancies. For some, engagement with genomic medicine leads to their own genetic diagnosis, whereas others discover their child’s syndrome resulted from a spontaneous mutation, shaping a new genetic subjectivity linked to whether they have the same gene variant or not. Their own genetic subjectivities (Buchbinder & Timmermans, 2011) and linked perceptions of genetic risk become tacit knowledge, often underlying their future reproductive decision‐making.

Parent’s imaginings of future pregnancies become biomedicalised and technologised, as they discuss potential reprogenetic technologies they could engage with. Their relationships to these technologies are complex and varying. Sometimes we see rejection and at other times they are sought out and consumed as a normative part of contemporary reproductive practices, where parents can make alliances with technoscience as a way to resist ableist ideologies that have become pervasive in medical discourse and practice around prenatal genetic testing. We can see this in Richard’s case, where he and his partner paid privately for NIPTs, rejecting amniocentesis because of perceived medical pressure towards selective termination. As other studies show, NIPTs can be an important aspect of parent’s quest for knowledge in the face of uncertainty (Boardman, 2017). Engaging with prenatal testing can be regarded as a mechanism to help parents feel prepared for the future, give them more time to come to terms with their child’s diagnosis and make arrangements for their future care needs (Shorey, 2023).

The decision to select against future children with NS through engagement with reprotech was highly emotive and difficult for parents to navigate. Like others have found, there can be significant discomfort around preconception and prenatal genetic testing and the idea of selective reproduction for those with lived experience of genetic conditions (Boardman, 2017). These technologies carry with them medicalised assumptions about the future life of their future child. Marred by ableism and stigma around birthing a disabled baby, access to reprotech is often controlled by medical gatekeepers, where termination of their disabled child is the only choice parents feel they are being supported to make, in our societies where disabled lives are devalued and often not deemed liveable (Goodley, 2018; Kafer, 2013). Society has little sympathy for those who choose to continue with such pregnancies or try for another baby knowing their future child might not be born healthy (Raspberry & Skinner, 2011).

It is not our intention in this article to rehearse debates around the ethics of genetic screening and selective reproduction in the context of disability. However the data suggest that there is still a need for genetic technologies and genetic counselling to be more responsive to the needs of the disability community. This includes providing options to families other than selective termination and recognising the value of genetic knowledge and early diagnosis for parents in their ability to plan for the future.

For all participants, their genetic subjectivities were entangled with care‐giving. Care, in parents’ accounts, was both a material practice involving the intimacies of giving medications, injections, therapies and meeting physical needs and an affective state involving responsibilities, compassion and nurturance to meet their child’s social and emotional needs (Vu Henry et al., 2021). Our data reveal how experiences of living with disability and the contours of care‐giving shift over time, taking parents through periods of crisis and stability. If health issues begin to stabilise or resolve, and the perceived care their disabled child required lessens, having the capacity to care for another child can become a more realistic possibility for these families.

For those who did attempt or go on to have subsequent pregnancies, their reproductive decisions were influenced not only by genetic knowledge and access to reprogenetic technologies but also by whether the family had sufficient emotional, financial, physical and temporal resources to care for another child. Thus, aligning with feminist disability studies perspectives to challenge the notion that reproductive decisions are purely individual choices. Our theoretical framework, combining insights from both medical sociology and disability studies sensitises us to how lived experience of disability shapes parent’s perceptions and values (Boardman, 2014). Ideas about ‘doing the right thing’ (Mendes et al., 2021) are entangled with parent’s perceived capacity to provide care, in a society where support is lacking across all domains and parents feel they must fight to get their child the resources they need. Thus, our analysis draws attention to the complexities of parenting in a world that values lives and bodies unequally and the messy affective spaces in which people live and make reproductive decisions, including the lack of support and resources to raise children with disabilities—which sits at the nexus of disability and reproduction (Casper, 2022).

While medicoscientifc rationalities for reprotech primarily centre on better detection of and mechanisms to select against disability in the early stages of human reproduction, our data suggest that this is not always the primary concern for parents of children diagnosed with a rare genetic condition. Their priority is being able access support and resources that allow them to care for their current and future children, regardless of their disability status. Thus, our efforts towards achieving reproductive justice (Roberts, 2015) should be directed towards providing these supports and resources. Alongside this, we need wider recognition of the value that reproductive technologies hold as informational tools that can be used not only for selection against future disability, but also to assist parents in making complex reproductive decisions by providing vital knowledge about the potential health of their future child(ren).

## AUTHOR CONTRIBUTIONS

**Catherine Coveney**: Conceptualization (lead); data curation (lead); formal analysis (lead); funding acquisition (lead); investigation (lead); methodology (lead); project administration (lead); supervision (lead); writing ‐ original draft (lead); writing – review & editing (lead). **Basma Salem**: Formal analysis (supporting); investigation (supporting); writing – review & editing (supporting).

## CONFLICT OF INTEREST STATEMENT

The authors declare no conflicts of interest.

## Data Availability

The data that support the findings of this study are available on request from the corresponding author. The data are not publicly available due to privacy or ethical restrictions.
